# Carbamazepine activates private self and viral reactive TCRs through a drug-permissive HLA-B cleft

**DOI:** 10.3389/fimmu.2026.1919927

**Published:** 2026-08-19

**Authors:** SuJin Hwang, Masahide Yano, Yura Jang, Maryam Khalaj, Elliot Mattson, Li Lu, Stephanie Kortchak, Jason Gorman, Montserrat Puig, Michael Norcross

**Affiliations:** 1 Division of Product Quality Research 4, Office of Product Quality Research, Office of Product Quality, Center for Drug Evaluation and Research, U.S. Food and Drug Administration, Silver Spring, MD, United States; 2 Division of Viral Products, Office of Vaccines Research and Review, Center for Biologics Evaluation and Research, U.S. Food and Drug Administration, Silver Spring, MD, United States

**Keywords:** carbamazepine, HLA-B*15:02, HLA-B*57:01, HLA-B*58:01, immunopeptidomics, Stevens-Johnson syndrome, T-cell receptor, toxic epidermal necrolysis

## Abstract

**Introduction:**

Carbamazepine (CBZ) can cause severe cutaneous adverse reactions, including Stevens-Johnson syndrome/toxic epidermal necrolysis, and is strongly associated with HLA risk alleles. However, HLA genotype alone incompletely predicts clinical risk. Adverse drug reactions depend on drug-peptide-HLA conformation, immuno-regulatory cell interactions and the presence of drug-specific TCR clonotypes in susceptible patients. An unresolved question is whether patients that develop a specific drug reaction harbor the same drug-specific TCR (public TCR) or generate patient unique TCRs (private TCR).

**Methods:**

To address this controversy we expressed four published CBZ-associated TCRs including two public and two private clonotypes, in TCR-null TG40 cells and tested activation using monoallelic 721.221 APCs expressing HLA-B*15:02, HLA-B*57:01, HLA-B*58:01, or HLA-B*58:01(TE→MA), as well as HLA-transgenic/MHC I-deficient murine splenic APCs. T-cell activation was quantified by PD-1, CD69, and 4-1BB, while LC-MS/MS immunopeptidomics/MAPPs was used to assess drug effects on the peptide repertoire. CBZ docking was applied to define drug-peptide-MHC-TCR interactions in a drug reactive peptide specific TCR.

**Results and discussion:**

Public TCRs showed no CBZ-dependent activation, whereas private TCRs displayed dose-dependent, HLA class I-dependent responses supported by HLA-B*15:02 and unexpectedly HLA-B*57:01, but not HLA-B*58:01. Amino acid substitutions to HLA-B*58:01 (T45-E46 to M45-A46) mapped drug presentation to restore CBZ responsiveness without altering HLA expression. CBZ did not detectably induce broad abacavir-like peptide motif alterations but amplified HIV Gag KF11 peptide-driven T cell activation in an allele- and mutation-dependent manner. Modeling predicted a candidate CBZ accessible region beneath the peptide near HLA Met67, HLA Tyr9, and a conserved CDR3α tyrosine providing a plausible structural rationale for allele and clonotype-dependent activation. These findings support a model in which CBZ potentiates binding of selected peptide-HLA-TCR interfaces governed by HLA cleft architecture and clonotype-level TCR sensitivity. This mechanism explains why HLA screening alone incompletely predicts CBZ-SJS/TEN risk and highlights peptide-HLA-TCR functional context as a potential determinant for improved risk assessment and mechanistic drug hypersensitivity testing.

## Graphical Abstract

**Figure:**
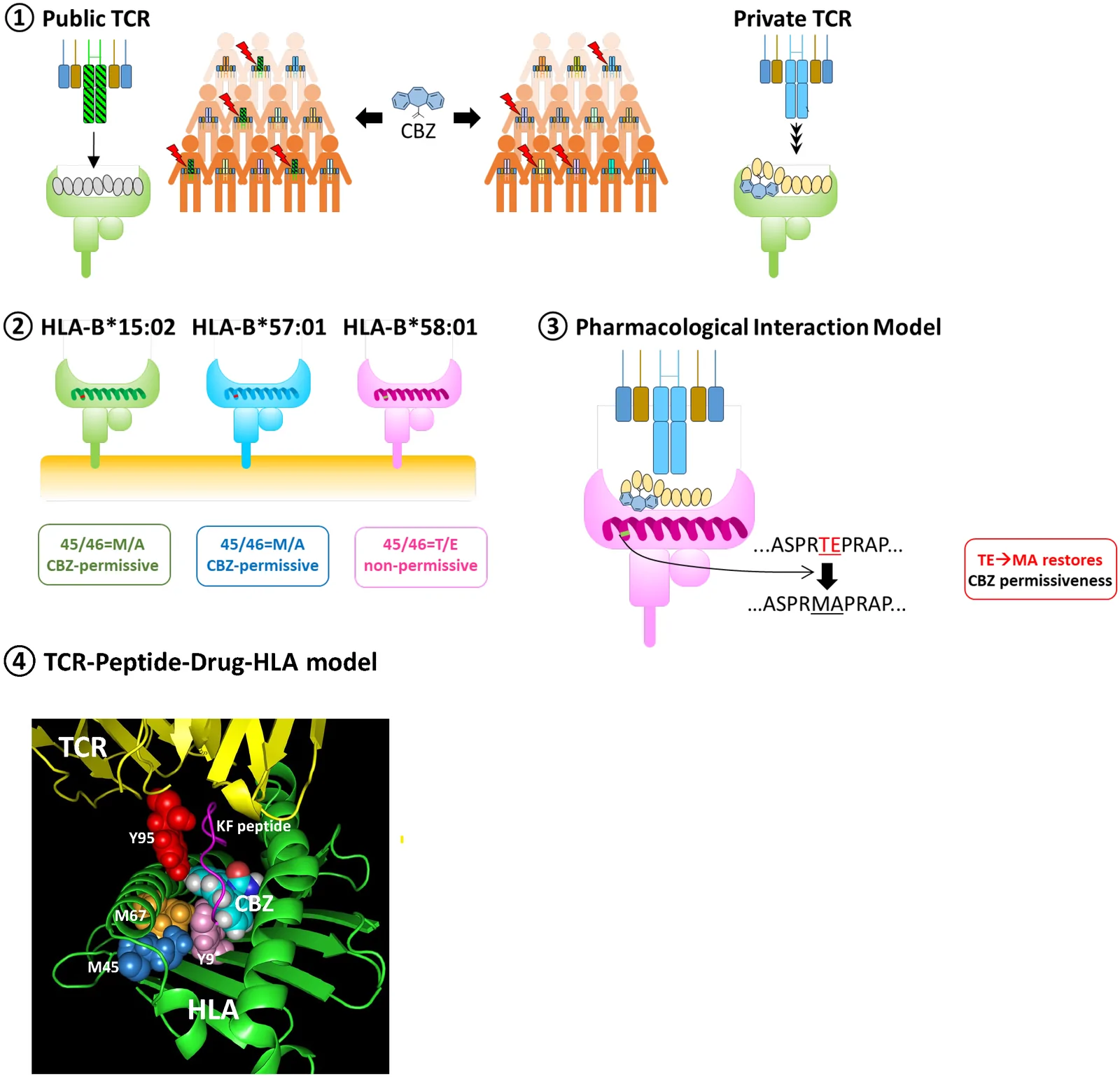
Public CBZ-associated TCRs showed weak self-reactivity but no CBZ-dependent activation, whereas private TCRs displayed basal self-peptide-HLA recognition that was selectively enhanced by CBZ. HLA-B*15:02 and HLA-B*57:01 share Met45/Ala46 at positions 45/46, creating a CBZ-permissive cleft environment, whereas HLA-B*58:01 contains Thr45/Glu46 and is non-permissive. Substitution of Thr45/Glu46 to Met45/Ala46 in HLA-B*58:01 restores CBZ responsiveness, supporting a role for this region in controlling drug permissiveness. Structural modeling using the TCR (yellow)-KF11 peptide (magenta)-HLA-B*57 (green) complex predicts a candidate sub-peptide CBZ (pale blue)-accessible region beneath the bound peptide (magenta), near HLA Met45 (sky blue), HLA Met67 (yellow‑orange), HLA Tyr9 (pink), and TCR Tyr95 (red). Alignment of the KF11-specific TCR with a structurally related private CBZ-reactive TCR suggests a shared CDR3α aromatic feature contributes to recognition of CBZ-modulated peptide-HLA complexes. Together, the model proposes that CBZ-dependent activation requires convergence of a permissive HLA-B cleft, compatible peptide conformation, and a sensitive private TCR clonotype.

## Introduction

Carbamazepine (CBZ) is broadly prescribed for epilepsy, trigeminal neuralgia, and mood disorders, yet it remains a leading culprit of severe cutaneous adverse reactions (SCARs), including Stevens–Johnson syndrome (SJS) and toxic epidermal necrolysis (TEN) (1–7). These reactions carry substantial morbidity and mortality and are driven by drug-specific T cells infiltrating the skin at disease onset (8–13). Recent prospective immunophenotyping of TEN blisters confirms that cytotoxic effector-memory CD8^+^ T cells dominate the inflammatory reaction, and deep TCR sequencing shows massive expansion of skin-recruited clonotypes that are also detectable in blood at presentation (14). The extent of these expansions correlates with clinical severity, underscoring a direct link between T-cell repertoire dynamics and disease pathophysiology (15–17). Clinically, defining the TCR and peptide-HLA features that distinguish pathogenic activation from tolerance is essential for improving risk prediction, diagnostic testing, and mechanistic modeling of CBZ-SJS/TEN beyond HLA genotyping alone.

HLA risk alleles are necessary but not sufficient. Strong associations between HLA class I alleles and CBZ hypersensitivity, most notably HLA-B*15:02 for CBZ-induced SJS/TEN and HLA-A*31:01 for broader CBZ reactions, have transformed clinical risk stratification in specific populations (18–29). HLA-B*57:01 has also recently been identified as a risk allele in European patients with CBZ reactions (30). However, the positive predictive value of these markers is modest (e.g., ~3% for HLA-B*15:02 in CBZ-SJS/TEN), implying that additional immunological determinants beyond HLA are essential for disease (31, 32). Using HLA-transgenic mice to study abacavir drug reactions in an animal model, we have defined key roles for CD4^+^ regulatory T cells in controlling HLA-linked T-cell reactivity to drugs through effects on antigen-presenting cells and CD8^+^ effector cells. Drug reactions involve type I/II interferon pathways and costimulatory molecule expression (33–36) to break tolerance. In addition to immunomodulatory mechanisms, a requirement for drug specific TCR clonotypes has been identified in patient cohorts. However, an outstanding question is whether patients with severe drug reactions to carbamazepine share the same TCR clonotype (“public”) or whether TCRs are unique to each patient (“Private”).

Early patient studies identified CBZ-reactive TCRs with evidence for shared public clonotypes, including restricted Vβ usage and recurrent CDR3 sequences that could mediate HLA-B*15:02-dependent cytotoxicity when re-expressed in T cells (15). Pan et al. further reported a paired public αβTCR enriched in blister cells and blood, with biochemical binding to CBZ/analogs and the capacity to transfer SCAR-like features into HLA-B*15:02 transgenic mice (16). Despite these seminal observations, larger HLA-diverse studies across culprit drugs reveal that TEN is characterized by massive expansions of unique private clonotypes with minimal sharing between patients. In a cohort spanning many HLA backgrounds and drugs, high-throughput TCR sequencing showed no shared CDR3 sequences across 15 TEN patients although patients were exposed to different drugs. Moreover, the degree to which the top skin clones circulated in blood at presentation correlated with the final extent of skin detachment, tying private clonal burden to clinical outcome. These data argue that individualized TCR usage is a major immunological hallmark of TEN (14). A recent report documented that CBZ-reactive T cells can be isolated from blood years after clinical SJS/TEN and demonstrated a private, rather than shared, repertoire between patients (17).

Public clonotypes in CBZ-SJS/TEN demonstrate that shared receptors can drive disease in defined HLA contexts; however, these sequences are not consistently observed across HLA-diverse populations and culprit drugs, may be detectable at low frequency outside disease, and often do not reflect the dominant patient-specific clonal expansions linked to clinical severity. In contrast, private clonotypes are shaped by an individual’s immune history and HLA-peptidome landscape and are most consistently enriched at sites of pathology and in blood at onset. From a TCR-biology perspective, this raises a central unresolved question: whether CBZ reactivity is best modeled by shared public receptors or patient-specific private clonotypes restricted by a set of peptide-HLA-TCR configurations that can differ between individuals. Resolving this question is clinically relevant because it may explain why some HLA-risk carriers remain tolerant whereas others develop severe CD8^+^ T-cell-mediated disease.

On this basis, to understand disease mechanisms and to set the groundwork for testing TCRs in HLA-transgenic models we re-examined a set of published public and private CBZ-reactive TCRs for drug and HLA specificity in TCR expressing *in vitro* assays. This approach allowed us to directly compare clonotype-dependent drug responsiveness, basal self-peptide reactivity, and HLA restriction under controlled antigen-presentation conditions. Together, our functional assays, immunopeptidomics, and modeling support a mechanism in which CBZ may potentiate or modulate selected peptide-HLA-TCR interfaces, strengthening the peptide-HLA-private TCR interface to amplify signaling, rather than inducing an abacavir-like altered peptide repertoire. Scientifically, this work provides a framework in which HLA cleft architecture, endogenous peptide conformation, and clonotype-level TCR sensitivity jointly determine CBZ-dependent T-cell activation. Clinically, it suggests that future risk assessment for CBZ-SJS/TEN may require functional evaluation of peptide-HLA-TCR interactions in addition to HLA genotype.

## Materials and methods

Comprehensive experimental details are provided in the **Supplementary Material**.

### Drugs and peptide

CBZ (Sigma-Aldrich, St. Louis, MO, United States) was dissolved in DMSO at indicated concentrations, aliquoted, and stored at -20°C until use. Abacavir was used as an altered-peptide-repertoire control where indicated. The HIV Gag KF11 peptide (KAFSPEVIPMF) (46, 47) was synthesized and purified to 98% purity at the Facility for Biotechnology Resources, Center for Biologics Evaluation and Research, U.S. Food and Drug Administration.

### TCR-TG40 cell lines

CD8α-expressing TG40 cells were transduced with retroviral pMSGV1 vectors encoding CBZ-associated TCRs with murine constant regions: Public TCR_1, Public TCR_2, Private TCR_1, and Private TCR_2 (See **Table 1**.). Viral particles were produced in GP2-293 packaging cells using Lipofectamine 3000. Transduced cells were screened by flow cytometry for murine TCRβ and human CD8 expression.

**Table 1:** CBZ-associated public and private TCRs and the KF11 HIV-specific control used in this study.

TCR	Source	Disease context	TRAV	TRAJ	CDR3α	TRBV	TRBJ	CDR3β	Reference
Public TCR_1	CBZ-SJS/TEN patient PBMC	SJS/TEN	TRAV9-2*01	TRAJ40*01	CAFISGTYKYIF	TRBV25-1*01	TRBJ2-1*01	CASSISGSYNEQFF	Ko *et al.* (15)
Public TCR_2	CBZ-SJS/TEN patient blister	SJS/TEN	TRAV12-1	TRAJ34	VFDNTDKLI	TRBV12-4	TRBJ2-2	ASSLAGELF	Pan *et al.* (16)
Private TCR_1	CBZ-TEN patient PBMC	TEN	TRAV21	TRAJ12	CAAKDGMDSSYKLIF	TRBV30	TRBJ 1-2	CAWLGAGKVDGYTF	Mifsud *et al.* (17)
Private TCR_2	CBZ-SJS patient PBMC	TEN	TRAV1-2	TRAJ20	CAAFGDYKLSF	TRBV27	TRBJ 2-3	CASSSLSGGWPDTQYF	Mifsud *et al.* (17)

### Monoallelic HLA-B APC lines and mutants

HLA-B*15:02, HLA-B*57:01, HLA-B*58:01, and HLA-B*58:01 mutants were expressed in HLA class I-deficient 721.221 cells using lentiviral vectors. HLA-B*58:01(TE→MA) denotes replacement of the HLA-B*58:01 residues Thr45/Glu46 with Met45/Ala46. Stable lines were selected with blasticidin, and HLA surface expression was confirmed by flow cytometry using anti-HLA-B/C antibodies.

### HLA-transgenic murine APCs

C57BL/6J mice expressing HLA-B*15:02 or HLA-B*57:01 alleles were generated as described previously. The HLA-B*57:01 transgene encoded human α1/α2 peptide-binding domains fused to murine α3, transmembrane, and cytoplasmic regions (35, 36), whereas the HLA-B*15:02 construct retained human α1-α3 domains. HLA expression and loss of murine H-2K^b^ on MHC I-deficient backgrounds were confirmed by flow cytometry. Animal studies were conducted under approved institutional protocols.

### T-cell activation assays

HLA-expressing APCs were treated with CBZ (25 or 50 µg/mL, unless otherwise indicated) and co-cultured with TCR-TG40 cells for 16-18 hours. T-cell activation was assessed by flow cytometry using PD-1, CD69, and 4-1BB. For KF11 assays, APCs were incubated overnight with KF11 ± CBZ or pulsed with KF11 for 6 hours, washed, and then co-cultured with KF11-specific T cells ± CBZ.

### Immunopeptidomics/MHC-associated peptide proteomics (MAPPS)

HLA-bound peptides were isolated by immunoaffinity purification with W6/32, acid-eluted, C18-desalted, fractionated, and analyzed by LC-MS/MS on an Orbitrap Fusion mass spectrometer. Peptides were identified with PEAKS Studio X+ using unspecific digestion and 5% FDR. Motif analyses focused on 9-mers. Peptide overlap across alleles was assessed in our laboratory and summarized using Venn diagrams.

### Structural comparison, docking, and visualization

HLA-B pocket comparisons used PDB 5VUD and 5VWH in PyMOL. Putative polar contacts around residues 45 and 67 were assessed using PyMOL distance and polar-contact tools. To identify plausible candidate CBZ binding sites, DiffDock (38) was first used for unbiased global site discovery across the TCR-peptide-HLA complex, followed by Rosetta (37) local refinement in the DiffDock-defined pocket. Molecular graphics were generated in PyMOL using the HLA-B*57-KF11-TCR structure (PDB 2YPL) and TCRα alignment templates as indicated.

### Statistical analysis

Data are presented as mean ± SD unless otherwise stated. Student’s t-test and one-way ANOVA were usedwhere appropriate, and P ≤ 0.05 was considered statistically significant. Analyses were performed using GraphPad Prism 10.

## Results

### Carbamazepine elicits HLA-B*15:02 restricted activation of private T-cell lines but not public T cell lines

The four CBZ-associated TCRs were selected from published CBZ-SJS/TEN studies to represent two previously proposed models of drug-reactive TCR biology: shared (“public”) clonotypes and patient-specific (“private”) clonotypes. Public TCR_1 (15) and Public TCR_2 (16) were selected because they were reported as recurrent or disease-enriched CBZ-associated receptors in HLA-B*15:02-positive CBZ-SJS/TEN cohorts. Private TCR_1 (17) and Private TCR_2 (17) were selected from paired single-cell TCR datasets of CBZ-reactive T cells from resolved SJS/TEN cases and represented dominant private CBZ-responsive clonotypes. These TCRs were not intended to represent the full diversity of all CBZ-SJS/TEN repertoires, but rather to provide a controlled side-by-side functional comparison of published public and private receptors under identical antigen-presentation conditions. To place the selected receptors in the context of published disease-associated TCR repertoires, we curated available CBZ-SJS/TEN TCR sequence data from prior studies and summarized V/J gene usage, CDR3 length, and recurrent CDR3 motifs. The selected public receptors mapped to previously reported shared/disease-enriched motifs, whereas the private receptors corresponded to dominant patient-specific CBZ-responsive clonotypes without a broadly shared CDR3 motif (**Table 1**).

Given the conflicting responses observed between public and private T cell lines in the context of CBZ treatment, we investigated whether carbamazepine (CBZ) directly activates T cells via specific HLA-B alleles. TCRs were constructed into retroviral vectors and transfected into the TCR negative TG40-hCD8 cell line (39). Flow cytometry confirmed hCD8, and TCR expression across engineered lines; Private_2 showed slightly higher murine TCR expression (**Supplementary Figure 1A**). HLA levels on B cell lines were broadly comparable, though 721.221_B*15:02 contained a small HLA-low subset (**Supplementary Figure 1B**).

**Figure 1:**
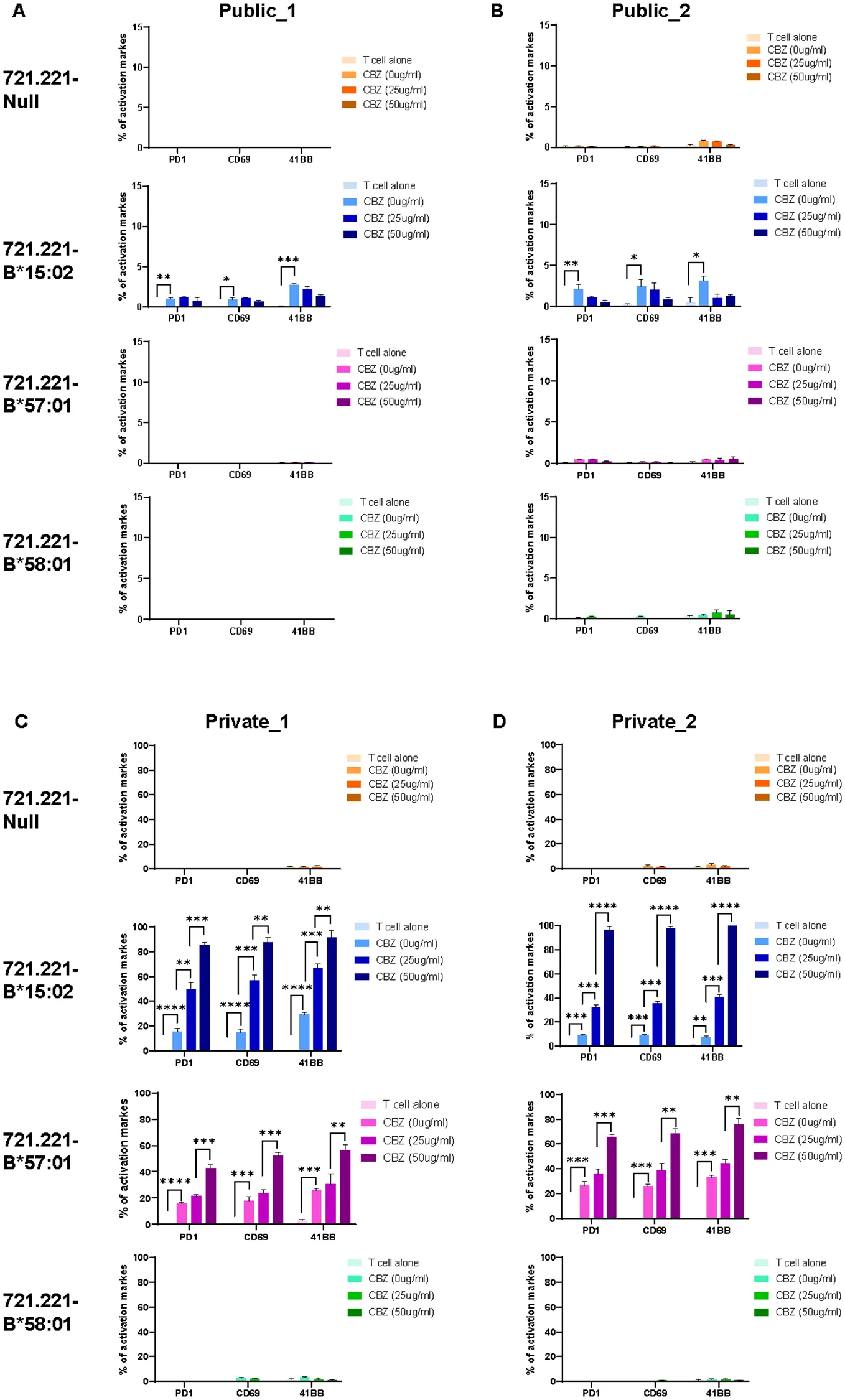
CBZ induces HLA-restricted activation in private, but not public, T-cell lines in a monoallelic APC system. **(A)** Public_1, **(B)** Public_2, **(C)** Private_1, and **(D)** Private_2 T-cell lines were cultured alone or co-cultured with monoallelic 721.221 APCs expressing HLA-null, HLA-B*15:02, HLA-B*57:01, or HLA-B*58:01. CBZ was added at 0 (no-drug/no-vehicle), 25, or 50 µg/mL, and activation was quantified by flow cytometry as the percentage of cells expressing PD-1, CD69, and 4-1BB. Bar graphs depict activation marker frequencies; statistical significance is indicated by asterisks. N = 3 (technical replicates), Bars show mean ± SD of technical replicate wells from one representative experiment; experiments were performed twice independently with similar results (biological replicates).

Public (Public_1/Public_2) and private (Private_1/Private_2) T-cell lines were cultured with mono-allelic 721.221 APCs expressing HLA-B*15:02, HLA-B*57:01, HLA-B*58:01, or HLA-null and treated with/without CBZ (0 (no-drug/no-vehicle), 25, 50 µg/mL). T-cell activation was assessed by PD-1, CD69, and 4-1BB upregulation, with αCD3 as a positive control (**Figures 1A-D**; **Supplementary Figure 2A-D**). T cells cultured alone or with HLA-null APCs showed no activation at all CBZ doses, whereas αCD3 induced robust marker upregulation, confirming assay sensitivity. Interestingly, public T-cell lines displayed weak basal activation with HLA-B*15:02 in the absence of CBZ, but CBZ did not enhance these responses (and slightly reduced them at higher doses); HLA-B*57:01 and HLA-B*58:01 did not elicit measurable activation at any CBZ concentration, indicating these public TCRs were not CBZ-reactive in this system (**Figures 1A, B**; **Supplementary Figure 2A, B**). In contrast, private T-cell lines showed stronger drug-independent basal activation with HLA-B*15:02 and similar or higher baseline activation with HLA-B*57:01. CBZ induced dose-dependent, HLA-restricted activation that was strongest with HLA-B*15:02 and moderate with HLA-B*57:01, but with no CBZ-driven activation with HLA-null or HLA-B*58:01 B cell lines (**Figures 1C,D**; **Supplementary Figure 2C,D**).

Notably, both public and private T-cell lines showed low-level drug-independent activation with HLA-B*15:02 APCs, consistent with weak recognition of endogenous self-peptide-HLA complexes. In addition, private T-cell lines exhibited baseline activation with HLA-B*57:01, indicating drug-independent low-affinity recognition of self-peptide–HLA complexes in the absence of CBZ (HLA-B*15:02 for both public and private lines; HLA-B*57:01 for private lines).

### HLA-transgenic murine APCs confirm CBZ-dependent activation in private T-cell lines

**Figure 2:**
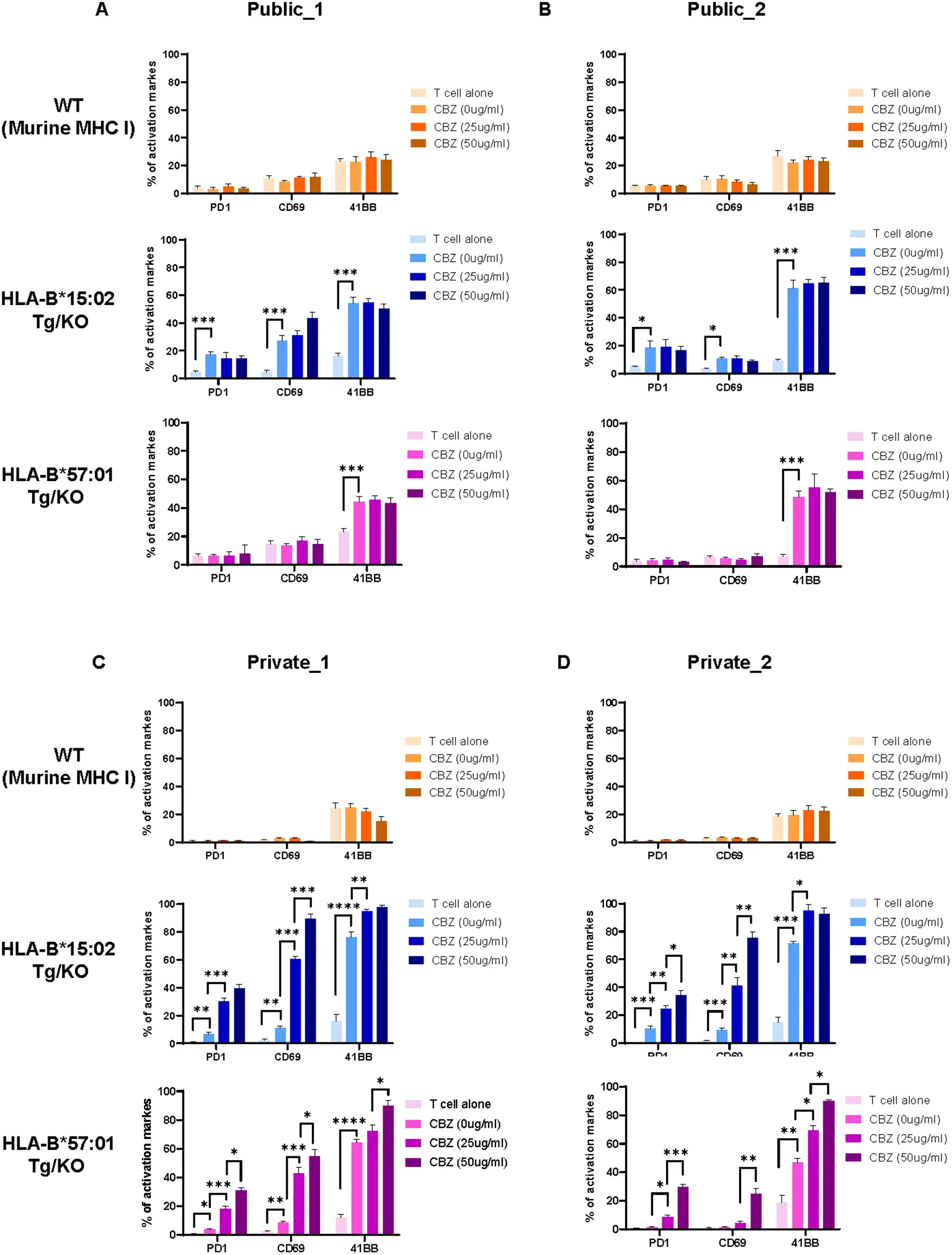
HLA-transgenic murine APCs support CBZ-dependent activation of private, but not public, T-cell lines. **(A)** Public_1, **(B)** Public_2, **(C)** Private_1, and **(D)** Private_2 T-cell lines were co-cultured with splenic APCs from wild-type mice or from HLA-B*15:02 Tg/MHC I-deficient or HLA-B*57:01 Tg/MHC I-deficient mice. CBZ was added at 0 (no drug/no vehicle), 25, or 50 µg/mL. T cells alone served as background control. T-cell activation was quantified as the percentage of cells expressing PD-1, CD69, and 4-1BB. N = 3 (technical replicates), Bars show mean ± SD of technical replicate wells from one representative experiment; experiments were performed twice independently with similar results (biological replicates).

To assess CBZ responsiveness under more physiological antigen-presentation conditions, we co-cultured public (Public_1/Public_2) and private (Private_1/Private_2) T-cell lines with splenic APCs from WT mice (murine MHC I only) or from HLA-B*15:02 Tg/MHC I KO and HLA-B*57:01 Tg/MHC I KO mice (human HLA class I in the absence of murine class I). Flow cytometry showed that splenic B cells expressed comparable levels of HLA class I across HLA-transgenic and HLA-transgenic/MHC I deficient mice (35, 36) and confirmed loss of murine class I (H-2K^b^) on the MHC I KO background (**Supplementary Figure 1D**). CBZ was added at 0 (no-drug/no-vehicle), 25, or 50 µg/mL, and PD-1, CD69, and 4-1BB expression was quantified by flow cytometry (**Figures 2A–D**; **Supplementary Figure 3A–D**). Because CBZ was dissolved in DMSO, we confirmed that DMSO vehicle alone did not increase activation-marker expression, despite the relatively high baseline observed in WT splenocyte co-cultures. Therefore, activation at 0 µg/mL CBZ in HLA-B*15:02 or HLA-B*57:01 conditions was interpreted as drug-independent, HLA-dependent basal reactivity only when it exceeded the matched WT/HLA-null background. CBZ-dependent activation was defined as increased marker expression above the matched no-drug/no-vehicle baseline within the same HLA condition.

Public T-cell lines displayed CBZ-independent basal activation when co-cultured with HLA-B*15:02 Tg/MHC I KO splenocytes, but CBZ did not increase PD-1, CD69, or 4-1BB expression. Public T-cell lines also showed low-level, CBZ-independent 4-1BB upregulation when co-cultured with HLA-B*57:01 Tg/MHC I KO splenocytes, however, CBZ did not augment this response, indicating no drug-dependent activation. This result contrasts with the report that Public TCR_2 induces drug induced skin reactions in an HLA-B*15:02 transgenic mouse reconstituted with TCR transfected T cells (16). Although we did not detect CBZ-dependent activation of the tested public TCRs in our system, both public TCRs exhibited CBZ-independent basal reactivity with HLA-B*15:02. This suggests that self-peptide-HLA recognition by transferred public TCR-expressing cells may have contributed to the SCAR-like phenotype reported in prior HLA-B15:02 transgenic mouse studies, independent of CBZ-specific signaling. WT splenocytes did not elicit measurable activation, indicating that the baseline responses required human HLA expression and were most consistent with weak self-peptide-HLA recognition rather than drug-driven signaling (**Figures 2A, B**; **Supplementary Figure 3A, B**). Private T-cell lines exhibited baseline activation with both HLA-B*15:02 and HLA-B*57:01 splenocyte APCs and, importantly, showed CBZ-enhanced activation that was stronger with HLA-B*15:02 and more modest with HLA-B*57:01 (**Figures 2C, D**; **Supplementary Figure 3C, D**).

In the *ex vivo* murine APC assay, CBZ did not elicit drug-dependent activation of the public T-cell lines when co-cultured with HLA-B*15:02 transgenic splenocytes. Nevertheless, splenocyte APCs supported CBZ-independent, allele-dependent basal activation in both public and private lines, with HLA-B*15:02 inducing upregulation of PD-1, CD69, and 4-1BB, whereas HLA-B*57:01 primarily increased 4-1BB.

### B-pocket micropolymorphisms at residues 45/46 distinguish CBZ-permissive (HLA-B*15:02/HLA-B*57:01) from non-permissive (HLA-B*58:01) alleles

**Figure 3:**
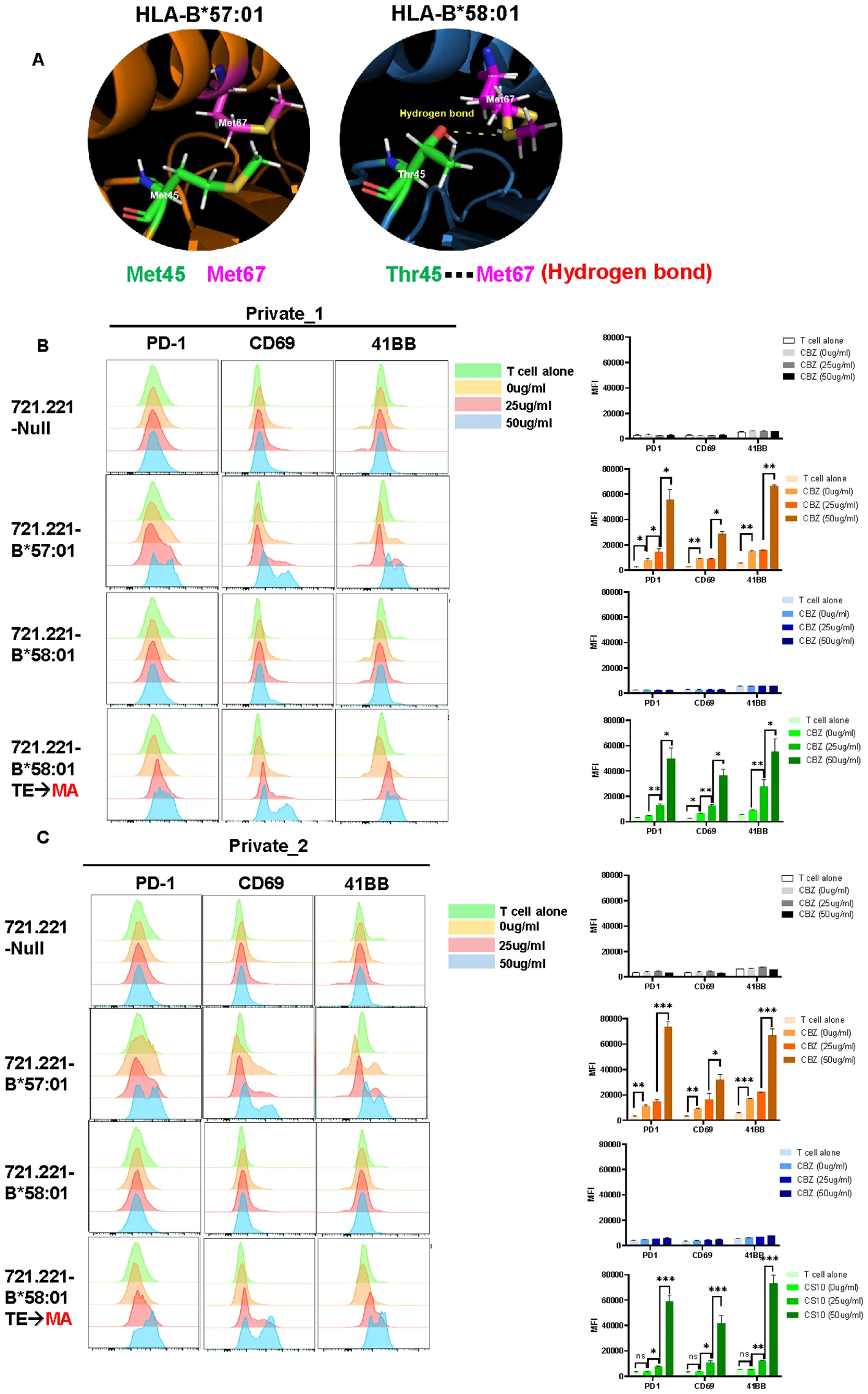
The HLA-B*58:01 TE→MA B-pocket substitution restores carbamazepine responsiveness in CBZ-specific private T-cell clones. **(A)** Structural close-ups of the B-pocket/P2 region showing residue 45 relative to Met67 in HLA-B*57:01 and HLA-B*58:01. In HLA-B*58:01, Thr45 forms a stabilizing hydrogen bond to the Met67 region, whereas Met45 in HLA-B*57:01 lacks this interaction. **(B)** Private_1 and **(C)** Private_2 T-cell lines were cultured alone or with 721.221 cells expressing HLA-null, HLA-B*57:01, HLA-B*58:01, or HLA-B*58:01(TE→MA) and treated with CBZ. PD-1, CD69, and 4-1BB expression were assessed by flow cytometry. N = 3 (technical replicates), Bars show mean ± SD of technical replicate wells from one representative experiment; experiments were performed twice independently with similar results (biological replicates).

In functional assays, we observed that HLA-B*15:02 and HLA-B*57:01, but not HLA-B*58:01, mediated a measurable response to CBZ in private T cell lines despite the high overall sequence similarity between HLA-B*57:01 and HLA-B*58:01. This pattern parallels the well-established CBZ reactivity associated with HLA-B*15:02 (18, 21, 31), suggesting that HLA-B*57:01 may share critical structural features with HLA-B*15:02 that are absent in HLA-B*58:01. We therefore sought to define the HLA determinants of this allele-specific CBZ responsiveness by identifying polymorphic residues conserved between HLA-B*15:02 and HLA-B*57:01 but divergent in HLA-B*58:01 within the α1/α2 peptide-binding platform. Based on IPD-IMGT/HLA comparison data, HLA-B*15:02:01:01, HLA-B*57:01:01:01 and HLA-B*58:01:01:01 are highly conserved across residues. HLA-B*57:01 and HLA-B*58:01 are nearly identical throughout the α1, α2 and α3 domains. Polymorphisms among HLA-B*15:02, HLA-B*57:01 and HLA-B*58:01 are mainly clustered within the α1/α2 peptide-binding platform (approximately positions 62-83), where HLA-B*57:01 and HLA-B*58:01 share multiple substitutions relative to HLA-B*15:02. Notably, comparison of B-pocket residues within the α1/α2 peptide-binding groove revealed greater similarity between HLA-B*15:02 and HLA-B*57:01 than with HLA-B*58:01 at positions 45 and 46: methionine at position 45 and alanine at position 46 are conserved in HLA-B*15:02 and HLA-B*57:01 but replaced by distinct residues in HLA-B*58:01 (**Supplementary Figure 4**). Structural comparison of the B-pocket region showed that HLA-B*57:01 contains a Met45-Met67 configuration lacking hydrogen-bonding constraints, whereas HLA-B*58:01 has Thr45-Met67 with a stabilizing hydrogen bond, indicating that residue 45 can alter local groove geometry and potentially the peptide-drug conformation (**Figure 3A**). These observations prompted us to examine the functional contribution of this difference, specifically on their impact on T-cell recognition and responsiveness.

### HLA-B*58:01(TE→MA) restores CBZ responsiveness in private T-cell lines

To determine whether structural features of HLA-B*58:01 can be modified to support CBZ dependent T-cell activation, we compared CBZ-specific CD8^+^ T-cell responses to 721.221 cells expressing Null, HLA-B*57:01, HLA-B*58:01 or mutant HLA-B*58:01 (TE**→**MA) alleles. Two private CBZ-specific CD8^+^ T-cell clones (Private_1 and Private_2) up-regulated PD-1, CD69 and 4-1BB only when co-cultured with HLA-B*57:01-expressing 721.221 cells in the absence and presence of CBZ as previously observed. Wild-type HLA-B*58:01 elicited negligible CBZ-dependent activation. In contrast, the TE**→**MA mutant of HLA-B*58:01 induced robust CBZ-dependent up-regulation of all three activation markers, approaching or exceeding the responses observed with HLA-B*57:01 (**Figures 3B, C**). CBZ independent self-peptide activation (CD69) with HLA-B*58:01 (MA) was observed in Private_1 TCR. The TE**→**MA substitution in HLA-B*58:01 induced CD69 upregulation and a modest 4-1BB signal. Surface staining of HLA-B on the different 721.221 transfectants showed comparable expression levels for wild-type and mutant molecules (**Supplementary Figure 1C**), indicating that the enhanced CBZ responsiveness of the MA mutant is not due to altered HLA abundance. This structural observation demonstrates that residues 45 and 46 (TE) are functionally critical for drug-HLA complex formation.

### Carbamazepine does not detectably induce abacavir-like remodeling of the HLA-B*15:02, HLA-B*57:01, and HLA-B*58:01 immunopeptidomes of wild-Type and mutant alleles

**Figure 4:**
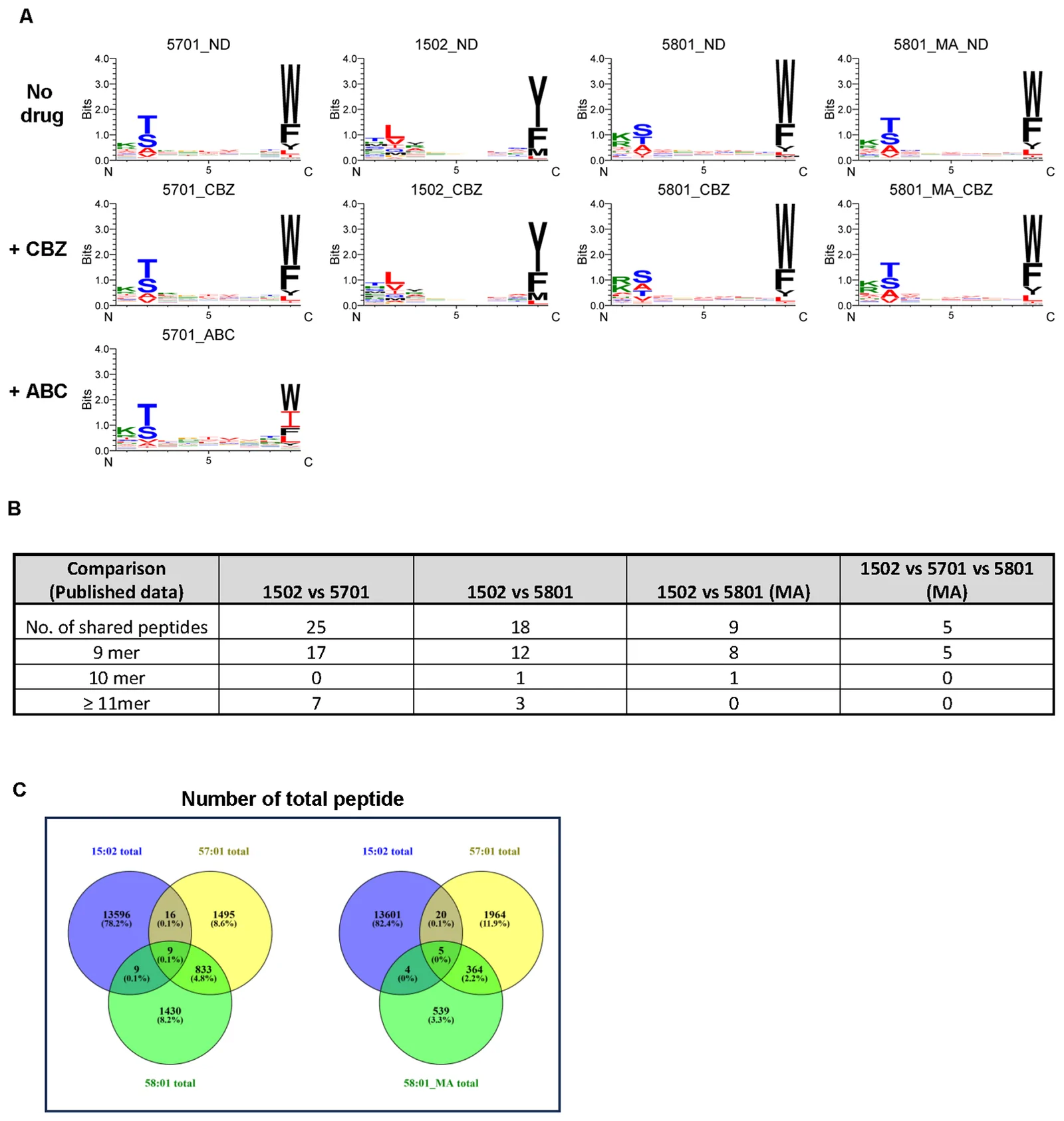
Carbamazepine does not induce abacavir-like immunopeptidome remodeling and reveals limited shared self-peptides across HLA-B allotypes. **(A)** Sequence logos of eluted 9-mer HLA class I ligands from monoallelic 721.221 cells expressing HLA-B*57:01, HLA-B*15:02, HLA-B*58:01, or HLA-B*58:01(TE→MA) cultured with no drug or CBZ. Abacavir treatment of HLA-B*57:01 cells served as a positive control for altered peptide repertoire. **(B)** Summary of shared peptides between indicated alleles, stratified by peptide length. **(C)** Venn diagrams depicting overlap of total peptide repertoires among HLA-B*15:02, HLA-B*57:01, and either HLA-B*58:01 or HLA-B*58:01(TE→MA).

To determine whether CBZ alters antigen presentation by risk and non-risk HLA-B alleles and whether the HLA-B*58:01 TE**→**MA substitution influences this process we performed LC-MS/MS immunopeptidomics on mono-allelic 721.221 cells expressing HLA-B*15:02, HLA-B*57:01, HLA-B*58:01, or HLA-B*58:01(MA) cultured ± CBZ or abacavir (ABC) (40, 41). Sequence logos confirmed expected allele-specific motifs under no-drug conditions (HLA-B*15:02 enriched for aromatic P9 residues; HLA-B*57:01/B*58:01 enriched for bulky hydrophobics), and ABC induced the characteristic HLA-B*57:01 C-terminal shift consistent with peptide alterations. In contrast, CBZ did not detectably alter peptide-binding motifs for any allele, including HLA-B*58:01(MA) (**Figure 4A**), arguing against an abacavir-like altered-peptide repertoire mechanism (42–45). HLA-B*58:01(MA) restored CBZ-dependent activation possibly by presenting drug responsive self-peptides. However, bulk immunopeptidomics may not detect rare, low-abundance, transient, or highly immunogenic ligands; therefore, subtle peptide-level changes or selected altered complexes cannot be excluded. These findings are most consistent with CBZ modulating selected peptide-HLA complexes rather than globally reshaping the immunopeptidome.

To explain how different HLAs present drug to drug specific T cells, we hypothesized there are shared peptides that are compatible for drug binding and T cell receptor recognition. Therefore, we next compared published self-immunopeptidomes for HLA-B*15:02, HLA-B*57:01, and HLA-B*58:01 with our MAPPs profiling of HLA-B*58:01(MA) to identify common peptides between HLAs (43, 44) (**Figures 4B, C**; **Supplementary Figure 5A**; **Supplementary Table 1**/Supplementary Repository data). Immunopeptidome analysis showed overlapping shared ligands between HLA-B*15:02, HLA-B*57:01and HLA-B*58:01 demonstrated by number of total peptides including 9-mers, 10-mers and longer peptides (**Figures 4B, C**; **Supplementary Figure 5B, C**). The HLA-B*58:01(TE**→**MA) mutant also showed overlap with HLA-B*15:02. This comparison demonstrated in principle that there are shared peptides between HLAs that are possible epitopes for drug specific TCRs. These data suggest that the MA45/46 substitution creates a CBZ-permissive cleft with a shared peptide that allows CBZ to modulate peptide-HLA complexes to drive drug-dependent activation.

### KF11 (HIV derived) peptide-driven T-cell activation is enhanced by carbamazepine in HLA-B*57:01 and HLA-B*58:01 (TE→MA), but not HLA-B*15:02

**Figure 5:**
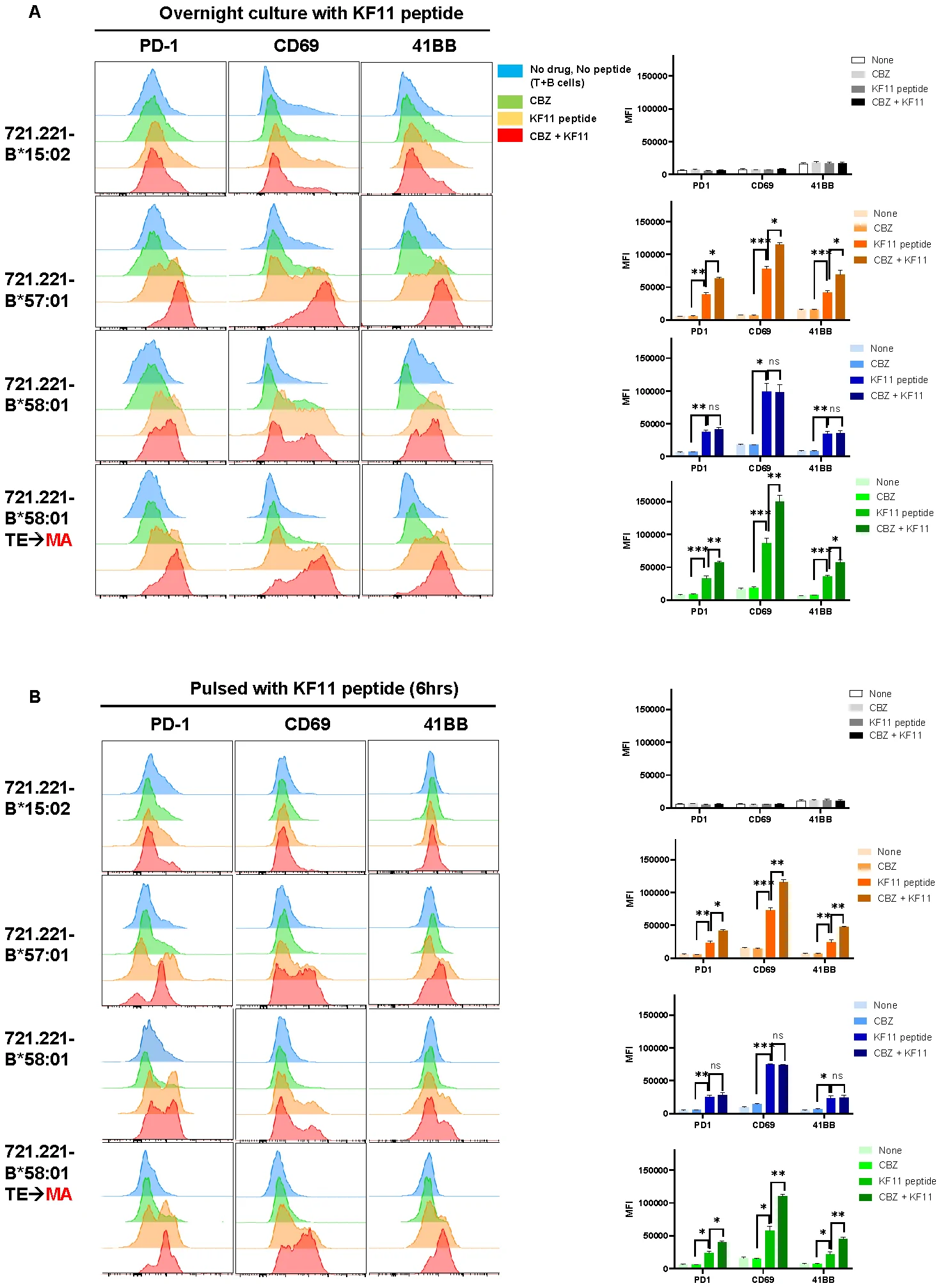
CBZ amplifies KF11 peptide-driven activation in an allele- and mutation-dependent manner. **(A)** Overnight co-culture and **(B)** 6-hour peptide pulse-wash assays were performed using 721.221 APCs expressing HLA-B*15:02, HLA-B*57:01, HLA-B*58:01, or HLA-B*58:01(TE→MA). Conditions included no treatment, CBZ (25 µg/mL), KF11 peptide (10 µM), or CBZ (25 µg/mL) + KF11 (10 µM). Histograms show PD-1, CD69, and 4-1BB expression; bar graphs show percentage of marker-positive cells. Representative flow-cytometry histogram overlays are shown with fluorescence intensity on a logarithmic x-axis and event counts on a linear y-axis normalized to mode using the FlowJo Modal normalization function. N = 3 (technical replicates), Bars show mean ± SD of technical replicate wells from one representative experiment; experiments were performed twice independently with similar results (biological replicates).

Because the endogenous self-peptides recognized by the CBZ-responsive private TCRs remain unknown self-peptides, and structural interrogation requires a defined peptide-HLA-TCR complex, we tested whether CBZ could modulate recognition of a well-characterized HLA-B*57:01-restricted peptide system (46, 47). We hypothesized that HLA-B*57:01-bound peptides, particularly longer ligands such as the HIV Gag KF11 (11-mer), could adopt a bulged conformation that creates a sub-peptide cavity permissive for CBZ. Accordingly, monoallelic 721.221 APCs expressing HLA-B*15:02, HLA-B*57:01, HLA-B*58:01 and HLA-B*58:01(MA) were incubated overnight with KF11 ± CBZ or pulsed with KF11 for 6 hours, washed, and then co-cultured with KF11-specific CD8^+^ T cells ± CBZ; activation was quantified by PD-1, CD69, and 4-1BB MFI (**Figures 5A, B**). CBZ alone did not activate the KF11-specific TCR in any HLA context, indicating that drug exposure was insufficient to bypass peptide specificity. KF11 induced minimal activation when presented by HLA-B15:02, but robust peptide-dependent activation when presented by HLA-B*57:01, HLA-B*58:01, or HLA-B*58:01(MA). Notably, addition of CBZ increased the magnitude of KF11-driven activation in an allele- and mutation-dependent manner, with the strongest enhancement observed for HLA-B*57:01 and HLA-B*58:01(MA), whereas wild-type HLA-B*58:01 showed or no CBZ-dependent augmentation despite presenting KF11. The same pattern persisted after peptide pulse-wash, arguing against increased peptide loading and supporting an effect of CBZ on signaling through a pre-formed peptide-HLA complex. Thus, peptide presentation alone was not sufficient for CBZ responsiveness; rather, CBZ enhancement required a permissive HLA cleft environment. These results support a model in which CBZ potentiates selected peptide-HLA-TCR interactions without overriding peptide specificity, although peptide titration and direct binding studies will be required to determine whether CBZ alters peptide EC50, TCR affinity, or functional avidity.

### Docking places CBZ beneath the peptide and near a shared CDR3α tyrosine

**Figure 6:**
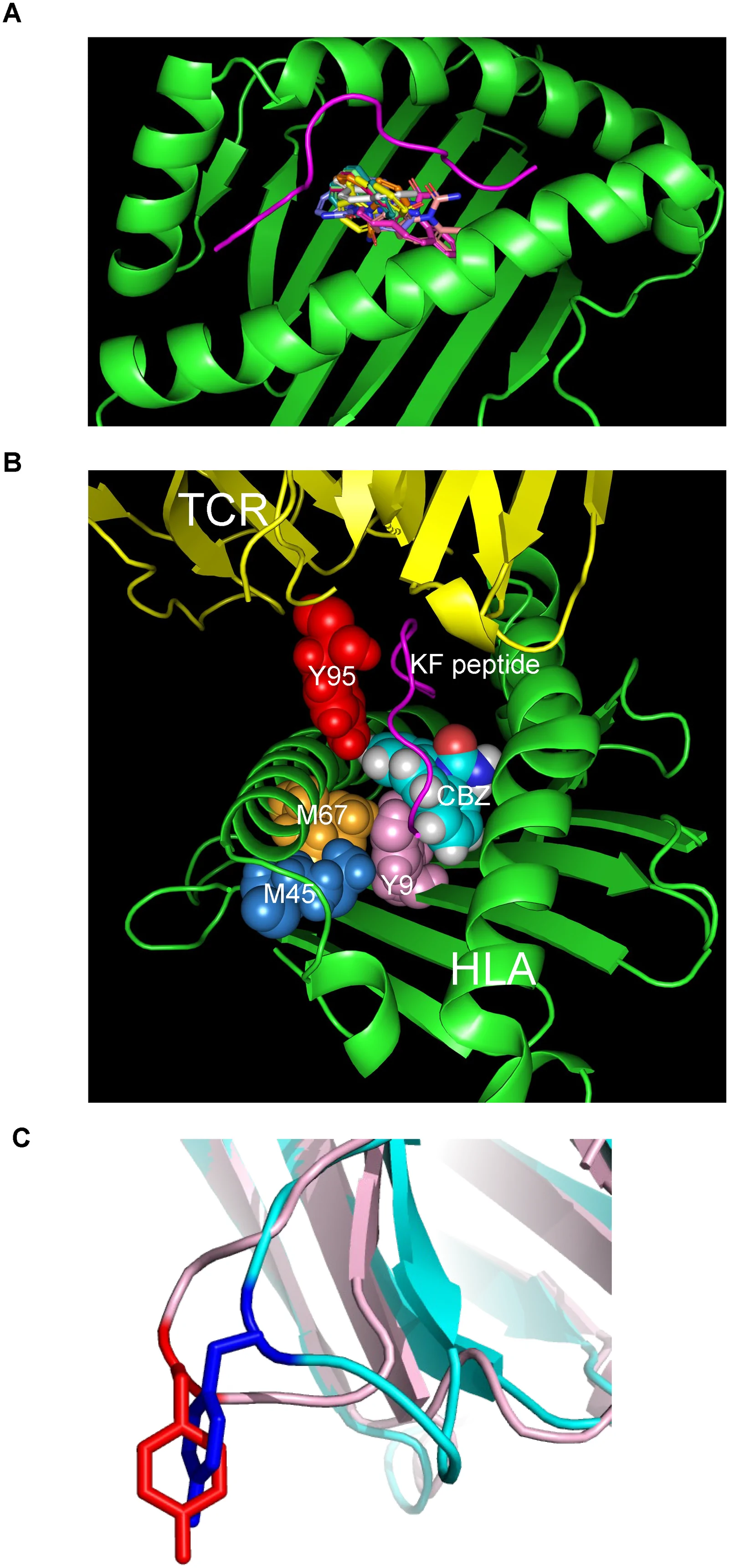
Structural modeling predicts sub-peptide CBZ binding and a shared CDR3α aromatic feature in CBZ-responsive TCRs. **(A)** DiffDock was used for global candidate-site discovery across the HLA-B*57:-KF11-TCR complex (2YPL), and the top predicted CBZ poses clustered within a region beneath the bound peptide in magenta. Multiple CBZ poses cluster beneath the bound peptide. **(B)** Rosetta local refinement within this region generated representative candidate poses in which CBZ is positioned near HLA Met67, HLA Tyr9, and TCR Tyr95. Close-up of the modeled TCR (yellow) -KF11 (magenta)-HLA-B*57 (green) complex highlighting CBZ (pale blue) under the peptide (magenta), adjacent to HLA Met45 (sky blue), HLA Met67 (yellow orange), and HLA Tyr9 (pink) and near TCR Tyr95 (red). **(C)** Structural alignment of TCR Vα domains from the KF11-specific TCR (blue) and CBZ-reactive Private_1 TCR (red) showing convergent positioning of a CDR3α tyrosine. These models are hypothesis-generating, identifying a plausible CBZ-accessible cavity beneath the peptide near HLA Met67, HLA Tyr9 and a CDR3a tyrosine but do not constitute direct evidence of CBZ binding or TCR-peptide-MHC stabilization.

The observation that a structurally defined TCR-peptide-HLA is responsive to CBZ provides a useful model to explore possible modes of CBZ engagement. Because the endogenous self-peptide recognized by the CBZ-responsive private TCRs remain unknown, we used the well-characterized HLA-B*57-KF11-TCR complex (PDB: 2YPL) to generate a hypothesis for how CBZ might interact with a permissive peptide-HLA-TCR interface. CBZ was docked onto this complex using DiffDock for unbiased candidate-site discovery (**Figure 6A**) followed by Rosetta for local refinement within the predicted region (**Figure 6B**). These analyses were used to identify plausible candidate CBZ-accessible regions and to provide a structural rationale for the functional data, rather than define an experimentally validated binding mode. Both approaches consistently positioned CBZ within a sub-peptide cavity beneath KF11 in the HLA groove at the TCR-peptide-MHC interface, supporting a model in which CBZ may stabilize a permissive peptide-HLA conformation and strengthen the peptide-HLA-TCR interaction. The top 10 DiffDock poses clustered within the same cavity beneath the peptide (**Figure 6A**), and Rosetta local refinement within this region (with conformational sampling and side-chain flexibility) yielded energetically optimized models (**Figure 6B**). In a representative Rosetta model, CBZ (pale blue) occupies the cavity adjacent to HLA Met67 (yellow-orange) and other pocket-lining residues and lies near TCR Tyr95 (red). Notably, the functionally mapped 45/46 region (Met45, sky blue) is not predicted to contact CBZ directly but instead resides beneath the peptide P2 region, consistent with an indirect steric/allosteric role in modulating access to the Met67-proximal pocket. suggesting that Met45 may indirectly influence local pocket geometry or access to a Met67-proximal region. HLA Tyr9 (pink) is also predicted to contact CBZ, potentially contributing to stabilization of the drug-bound complex (**Figure 6B**). To relate this binding mode to CBZ-reactive private receptors, we aligned a structurally similar TCRα template selected based on Vα 21.01 and CDR3α (MDSSYKLIF) similarity to Private_1 (blue, PDB: 3VXR) to the KF11 TCRα (red, PDB: 2YPL). Despite distinct Vα usage, the alignment revealed a convergently positioned CDR3α tyrosine (KF Tyr95 and the corresponding private TCR tyrosine) projecting into a similar spatial location over the predicted CBZ pocket (**Figure 6C**), consistent with a shared aromatic feature capable of sensing CBZ-stabilized peptide-HLA complexes. Because these models are computational and were not validated by direct binding or structural assays, they should be interpreted as a plausible structural rationale rather than definitive evidence of CBZ binding. Thus, the modeling supports a testable hypothesis in which HLA-B micropolymorphisms shape a CBZ-permissive peptide-HLA conformation.

## Discussion

A fundamental unresolved issue in CBZ-induced SJS/TEN is the low positive predictive value of strong HLA associations such as HLA-B*15:02, implying that additional determinants beyond HLA genotype, including immune tolerance, immune regulation, peptide presentation, and TCR repertoire are required for disease. Prior work has demonstrated that TEN lesions are dominated by cytotoxic effector-memory CD8^+^ T cells with marked clonal expansions that correlate with clinical severity, linking disease development to the responding TCR repertoire (9, 10, 48–51). Although public clonotypes have been reported to mediate CBZ- and HLA-B*15:02-dependent activation *in vitro* (15) and to transfer SCAR-like phenotypes in HLA-B*15:02 transgenic mice (16), CBZ-SJS/TEN is frequently characterized by private, patient-specific expansions with limited inter-individual sharing (14, 17). The clinical and scientific importance of our study is that it directly compares these public and private TCR models under standardized antigen-presentation conditions and shows that CBZ responsiveness is governed not simply by HLA genotype, but by the integrated peptide-HLA-TCR interface.

In this study, private TCR-transfected T-cell lines showed clear dose-dependent CBZ-induced upregulation of PD-1, CD69, and 4-1BB, whereas public TCR lines showed no meaningful CBZ-dependent activation across HLA contexts. The low-level activation observed in public lines with HLA-B*15:02 in the absence of CBZ is most consistent with weak recognition of endogenous self-peptide–HLA complexes rather than drug-driven signaling. The lack of CBZ-dependent activation by the tested public TCRs was unexpected given prior reports of public CBZ-associated clonotypes. However, our findings should be interpreted in the context of the experimental systems used. The original public TCR studies differed from the present work in APC type, source of peptide repertoire, TCR expression system, disease stage and tissue source of the TCRs, and functional readouts. In our monoallelic 721.221 and HLA-transgenic splenocyte APC systems, the public TCRs showed either no activation or weak CBZ-independent basal activation but did not display reproducible CBZ-dependent enhancement. Thus, our data do not exclude public TCR pathogenicity in all contexts; rather, they indicate that CBZ responsiveness by these public TCRs is not reproduced under our defined APC and reporter-cell conditions. Public TCR sequences detected in patient samples may also include self-reactive or bystander clonotypes expanded or recruited in inflamed tissue, including tissue-resident memory bystander cells activated by local cytokines together with self-antigen (52, 53). This reinforces the possibility that CBZ-dependent activation requires a specific combination of peptide repertoire, HLA conformation, and TCR activation threshold, and that private clonotypes may provide a more robust substrate for modeling patient-specific CBZ reactivity. In contrast, private TCRs exhibited measurable drug-independent baseline activation with HLA-B*15:02 and HLA-B*57:01, and unlike the public TCRs, these responses were further amplified by CBZ. Together, these data support a model in which CBZ preferentially enhances signaling only for specific peptide-HLA-TCR triads (17), helping explain why some clonotypes display basal self-peptide reactivity without drug responsiveness and why only a subset of HLA-risk individuals may develop pathogenic CD8^+^ T-cell activation. Although PD-1, CD69, and 4-1BB upregulation provided sensitive readouts of TCR activation, this reporter system does not fully capture cytotoxic effector function. CD107a degranulation and intracellular granzyme B/perforin staining were not performed because TG40 cells are TCR-null murine reporter cells rather than differentiated cytotoxic CD8^+^ T cells, and therefore may not faithfully model granule release, granulysin production, or keratinocyte killing. Future studies should evaluate private CBZ-responsive TCRs in primary human CD8^+^ T cells or patient-derived clones using CD107a, granzyme B, perforin, granulysin, and direct cytotoxicity assays against tissue-relevant target cells.

Unexpectedly, CBZ-responsive private TCRs were activated not only by HLA-B*15:02, but also when CBZ was presented by HLA-B*57:01, suggesting a shared permissive cleft environment and overlapping TCR-facing peptide-HLA surface. In contrast, the closely related HLA-B*58:01 did not support CBZ-dependent activation, indicating that peptide presentation alone is insufficient and that subtle HLA micropolymorphisms can determine whether a peptide-HLA complex is both TCR-engaging and CBZ-permissive. We identified B-pocket residues 45/46 as candidate determinants: HLA-B*15:02 and HLA-B*57:01 share Met45/Ala46, whereas HLA-B*58:01 encodes Thr45/Glu46. Consistent with this, a targeted TE→MA substitution in HLA-B*58:01 restored robust CBZ-dependent activation in private T-cell lines without altering HLA surface expression.

To determine whether shared self-peptides could provide a basis for cross-recognition, we compared published immunopeptidomes with our in-house MAPPs data across HLA-B*15:02, HLA-B*57:01, HLA-B*58:01, and HLA-B*58:01(MA). These analyses identified overlapping self-peptides across distinct HLA-B allotypes, suggesting potential candidate ligands for CBZ-reactive TCRs. However, the presence of a shared peptide sequence does not necessarily indicate that the peptide is displayed in an equivalent TCR-facing conformation. The same peptide may adopt different registers, bulged conformations, surface exposure, stability, or abundance depending on the HLA cleft. Thus, HLA-B*58:01 may present overlapping self-peptides but fail to induce basal self-reactivity because the resulting peptide-HLA complex does not engage the private TCR above the activation threshold.

These findings suggest that CBZ permissiveness and basal self-reactivity are related but separable properties. HLA-B*15:02 and HLA-B*57:01 may present shared or structurally similar self-peptides with sufficient stability, abundance, and TCR-facing geometry to support low-level tonic TCR engagement in the absence of drug. By contrast, wild-type HLA-B*58:01 may present related peptides but display them in a nonpermissive conformation and, through its Thr45/Glu46 configuration, constrain the local B-pocket/sub-peptide environment required for productive CBZ binding. Conversion of HLA-B*58:01 to the MA45/46 configuration may restore the CBZ-accessible cleft and allow drug-dependent enhancement of selected peptide-HLA complexes, while not necessarily restoring the precise peptide affinity, surface density, or conformation required for CBZ-independent basal self-reactivity. Identification and direct loading of the activating self-peptide(s) will be required to determine whether the same peptide mediates basal and CBZ-dependent recognition across these HLA contexts.

Mechanistically, our findings are more consistent with a stabilization/p-i–like mechanism than with the altered peptide repertoire model exemplified by abacavir (54). Abacavir binds HLA-B*57:01 and reshapes the self-peptidome to generate “altered self” ligands that activate polyclonal CD8^+^ T cells (43). In our immunopeptidomics, abacavir produced the expected HLA-B*57:01 motif shift, whereas CBZ did not detectably induce abacavir-like motif remodeling on HLA-B*57:01 or with the other HLAs tested (HLA-B*15:02, HLA-B*58:01, or HLA-B*58:01-MA) consistent with other studies (17, 41). Thus, CBZ biology in this system is better captured by binding to a permissive cleft site on pre-formed peptide-HLA complexes, stabilizing select conformations rather than driving bulk peptidome remodeling (17). Because CBZ responsiveness may depend on permissive peptide-HLA conformations, we speculated that longer ligands could bulge within the class I groove and generate a sub-peptide cavity accessible to CBZ, although shorter peptides may also support CBZ binding depending on their conformation.

To test this concept in a defined peptide system, we examined whether CBZ modulates recognition of the HLA-B*57:01-restricted HIV Gag KF11 epitope, an 11-mer peptide, using a KF11-specific CD8^+^ T-cell clone. CBZ alone did not activate the KF11-specific TCR, but CBZ enhanced KF11-driven activation most clearly when KF11 was presented by HLA-B*57:01 and HLA-B*58:01(MA), with little effect in wild-type HLA-B*58:01. This enhancement persisted after peptide pulse-washout and remained strictly peptide-dependent, indicating that CBZ does not increase peptide loading or bypass peptide specificity, but instead acts as an allele- and mutation-dependent amplifier of peptide-driven signaling. The finding that HLA-B*58:01(MA) enhances CBZ responsiveness with the KF11 peptide supports a mechanism in which Met45/46 affects CBZ-binding-site permissiveness rather than simply altering the peptide specificity of the TCR.

Structural modeling provides a plausible, but hypothesis-generating, explanation for the allele difference and the MA rescue. In HLA-B*58:01, a predicted Thr45-Met67 hydrogen bond, absent in HLA-B*57:01, may constrain local groove architecture and limit formation or accessibility of a sub-peptide cavity. Across docking solutions, candidate CBZ is predicted to bind beneath the peptide, and position near the TCR-peptide-MHC interface in proximity to TCR Tyr95, supporting a possible direct stabilization of the interface. In this model, Met45 may allosterically influence Met67 and help stabilize a Tyr9 rotamer that closely contacts CBZ, whereas Thr45-mediated hydrogen bonding to Met67 may constrain this configuration and interfere with drug binding. TCR structural overlays further revealed a shared recognition hotspot, with a similarly positioned CDR3α tyrosine in the KF11-specific TCR and a Vα/CDR3 structural homolog of the CBZ-reactive private TCR_1.

This recurrent aromatic feature may sense or stabilize the CBZ-modified peptide-HLA interface. Collectively, these interactions could strengthen TCR engagement by altering affinity, bond lifetime under force, and downstream contributions from coreceptor and integrin engagement (55). Although DiffDock and Rosetta placed CBZ in a sub-peptide cavity near the peptide-HLA-TCR interface, these computational models should be interpreted as hypothesis-generating rather than definitive evidence of drug-induced stabilization. We did not directly measure TCR-peptide-MHC binding affinity, tetramer off-rates, or bond lifetime in the presence of CBZ. Thus, our structural model is supported indirectly by the functional HLA-B*58:01 TE→MA rescue, the absence of abacavir-like immunopeptidome alteration, and the CBZ-dependent enhancement of KF11-driven activation. Direct validation will require using soluble TCR-peptide-MHC binding and stability assays, TCR/HLA-pocket mutagenesis, ultimately 3D structure determination.

Our study has several limitations. The number of TCRs tested remains small relative to patient repertoire diversity, and our findings should be extended to additional private clonotypes from independent CBZ-SJS/TEN cases and HLA backgrounds. Activation-marker upregulation provides a sensitive functional readout but does not fully capture cytotoxic effector function, keratinocyte injury, or tissue-level inflammatory amplification. Immunopeptidomics depth may miss rare but immunodominant ligands, and the precise self-peptides required for private TCR activation remain unidentified. Finally, although docking and Rosetta modeling support a sub-peptide CBZ-binding model, direct structural or biophysical evidence is needed to confirm CBZ positioning and its effect on peptide-HLA-TCR stability. Future studies should define minimal activating self-peptides for private CBZ-reactive TCRs, validate permissive HLA and TCR residues, test tissue-relevant APCs including keratinocytes and skin APC subsets, and expand private TCR sampling across patients to determine the generalizability of HLA-B*57:01-permissive CBZ presentation.

Overall, our data support a framework in which CBZ engages a structurally permissive environment within the HLA class I cleft shared by HLA-B*15:02 and HLA-B*57:01, stabilizing otherwise weak self-peptide-HLA-TCR interactions. This model accommodates the observed clonotype dependence, allele selectivity, separation of CBZ permissiveness from basal self-reactivity, lack of abacavir-like peptidome remodeling, and CBZ-mediated amplification of peptide-driven antiviral TCR signaling without overriding peptide specificity. Clinically, these findings help explain why HLA genotype alone incompletely predicts CBZ-SJS/TEN risk and suggest that future risk assessment may require functional consideration of HLA cleft architecture, peptide context, and clonotype-level TCR sensitivity. The ability of CBZ to enhance KF11-specific CD8^+^ T-cell activation also suggests that CBZ can modulate antiviral memory T-cell responses in a peptide- and HLA-dependent manner; however, any clinical relevance to antiviral immunity remains speculative and requires dedicated *in vivo* investigation.

## Data Availability

The mass spectrometry proteomics data have been deposited to the ProteomeXchange Consortium via the PRIDE partner repository with the dataset identifier PXD082505.
