# Supplementary material for: Carbamazepine activates private self and viral reactive TCRs through a drug-permissive HLA-B cleft
Source: Front Immunol. 2026 Aug 19;17:1919927. doi: 10.3389/fimmu.2026.1919927 (PMC13536285; doi:10.3389/fimmu.2026.1919927)
Supplement: Supplementary file 1 [file Table2.docx]

**Supplementary Material**

**Carbamazepine Activates Private Self and Viral Reactive TCRs Through a Drug-Permissive HLA-B Cleft**

**Supplementary Methods**

**Drug**

Carbamazepine (CBZ) used for *in vitro* and *in vivo* administration was obtained from Sigma-Aldrich (St. Louis, MO). The drug was dissolved in Dimethyl sulfoxide (DMSO) (Sigma-Aldrich) at desired concentrations and kept aliquoted at -20°C until use.

**Carbamazepine -specific T cell receptors on TG40 cell line construction**

The CD8α-expressing TG40 cell (1) was initially constructed by Dr. Takamasa Ueno at Kumamoto University, Kumamoto, Japan, and kindly shared by Dr. Hiroyuki Kishi at University of Toyama, Toyama, Japan. To generate Carbamazepine -specific T cell receptors TCR on CD8:TG-40 cells, we prepared a retroviral mammalian TCR expression vector pMSGV1 (GeneScript, Piscataway, NJ) with Public TCR_1 (TCR VA22-CAFISGTYKYIF/TCR VB11-CASSISGSYNEQFF)) (2) , Public TCR_2 (TCR VA- VFDNTDKLI/TCRVB- ASSLAGELF) (3) , Private TCR_1 (E10056: TCR VA- CAAKDGMDSSYKLIF/TCRVB- CAWLGAGKVDGYTF) (4) and Private TCR-2 (E10630: (TCR VA- CAAFGDYKLSF/TCRVB- CASSSLSGGWPDTQYF)) (4) with a mouse TCR constant region, and transfected to a GP2-293 packaging cell (Takara Bio, Mountain View, CA) using Lipofectamine 3000 by following the manufacturer’s instruction (ThermoFisher, Waltham, MA). Viral supernatant was harvested from the packaging cell. Viral supernatant stocks were used for subsequent transduction experiments with CD8- expressing TG40 cells. Following transduction, the positive populations for mouse TCR constant region were screened by anti-mouse TCR β chain antibody (H57-597) (BioLegend, San Diego, CA), hCD8 and HLA-B/C at the Flow Cytometry Core, CBER in FDA.

**HLA specific B cell cell line construction**

We established a membrane-bounded (m) HLA-B*15:02, 57:01 and 58:01 -expressing cell line: 721.221: mHLA-B*15:02, 57:01 and 58:01 by transducing ATCC CRL-1855™ (721.221, ATCC) with lentiviral mammalian-expressing vector pcDNA3.1-carrying mHLA-B*15:02, 57:01 and 58:01.

**Generation of HLA-B*58:01 and HLA-B*58:01 MA, transgenic B cells**

Lentiviral vectors encoding HLA-B*58:01, HLA-B*58:01 MA, were generated by transfecting the respective constructs into 293FT cells (Thermo Fisher, R70007) following the manufacturer’s instructions. The resulting viral particles were used to transduce the HLA Class I-deficient B cell line 721.221 (ATCC CRL-1855™). Cells were initially seeded at 0.2 × 10⁶ cells/mL in a 75 cm² flask and maintained at a density of 1-1.5 × 10⁶ cells/mL. For each construct, 2 × 10⁶ 721.221 cells were transduced with the corresponding lentiviral particles. Transduced cells were selected with blasticidin for 8 weeks, and expression of the transgenes was confirmed by flow cytometry.

**Measurement of T cell activation**

The HLA-expressing B cell lines (721.221 Null, 721.221_B*15:02, 721.221_B*57:01 and 721.221_B*58:01) and HLA transgenic splenocytes (HLA-B*15:02, HLA-B*57:01) were treated with carbamazepine (25, 50 µg/mL) or abacavir (10 µg/mL), and co-cultured with the CBZ specific T cell lines (Public TCR 1, 2, Private TCR 1, 2, KF11-hCD8:TG40 cells for ~16-18 hours. Activation of T cells were evaluated by measuring activation markers (e.g., PD-1, C69 and (4-1BB)) expression level by flow cytometry. In T-cell activation assays, TCR-TG40 cells cultured alone served as the biological negative activation control. HLA-null B cell line and mouse spleen co-cultures without drug served as background APC controls, and αCD3 stimulation served as the positive activation control. Activation-marker expressions for PD-1, CD69, and 4-1BB was interpreted relative to these biological baseline controls. Matched isotype-control staining for PD-1, CD69, and 4-1BB were comparable to the non-activated T-cell-alone baseline, confirming minimal nonspecific antibody staining and supporting use of the T-cell-alone condition as the negative activation reference. Vehicle-control wells received the same volume of DMSO used for CBZ-treated wells (5 µL/well). DMSO alone did not increase PD-1, CD69, or 4-1BB expression compared with untreated T-cell-alone or HLA-null APC (WT APC (Murine MHC I) coculture controls.

**Peptide synthesis and drug preparation**

The following synthetic peptide (KF11: KAFSPEVIPMF). were synthesized and purified to 98% purity at Facility for Biotechnology Resources at Center Biologics Evaluation and Research (CBER) at FDA.

**Cell maintenance**

The established cell lines were maintained in the following conditions: Cell culture medium RMPI 1640 medium (ThermoFisher) supplemented with 10% fetal bovine serum (FBS) (Atlanta Biologicals, Flowery Branch, GA), 1% penicillin/streptomycin, 1% Sodium Pyruvate and 2mM L-glutamine (ThermoFisher). All cell lines were maintained at 37°C with 5% CO2.

**Mice**

C57BL/6J mice expressing the HLA-B*15:02 or HLA-B*57:01 alleles were generated based on the chimeric HLA transgene 5. HLA‑B*57:01 transgene encoded a chimeric class I heavy chain comprising the human α1/α2 peptide‑binding domains fused to the murine α3 domain and murine transmembrane/cytoplasmic regions, whereas the HLA‑B*15:02 construct retained fully human α1-α3 domains. All animal studies complied with the Institutional Animal Care and Use Committee welfare guidelines of the US Food and Drug Administration (FDA). Expression of HLA on mouse cells were confirmed by flow cytometry using anti HLA-B/C (clone number) anti MHC Class I (H-2K^b^) (Fisher Scientific; AF6-88.5.5.3) Monoclonal Antibody.

**Immunopeptidomics**

A total of 5-20 x 10^6^ cells were lysed by sonication on ice in lysis buffer, 20 mM Tris (pH 8), 1 mM EDTA, 100 mM NaCl, 1% Triton X-100, 60 mM octyl β-D-glucopyranoside, 1x protease inhibitor, and 50 unit/mL of benzonase. The cell lysates were centrifuged at 12,000 rpm for 20 min at 4°C. The supernatant was mixed with CaptivA rProtein A affinity resin and anti–HLA-ABC W6/32 antibody and incubated with gentle rotation at 4°C overnight. After washing, the beads were transferred onto in-house-made C_18_ StageTips, and the HLA-bound peptides were eluted with 10% acetic acid. Peptides were collected using a step gradient of 20%, 40%, and 60% acetonitrile in 0.1% formic acid (FA). All eluates were combined, dried completely, and stored at –80°C until use.

The peptides were reconstituted in 0.1% FA and analyzed using an Ultimate 3000 RSLCnano system coupled to a Fusion Orbitrap mass spectrometer, equipped with a trap column (Acclaim PepMap C_18_, 5 µm, 100 Å, 20 mm x 100 µm) and an analytical column (Easy-Spray PepMap C_18_, 3 µm, 100 Å, 15 cm x 75 µm), using a linear gradient at a flow rate of 250 nL/min., following standard settings at the FDA's Facility for Biotechnology Resources. Data acquisition was performed in data-dependent acquisition mode. Precursor mass scans were acquired at a resolution of 60,000, followed by MS/MS scans at a resolution of 15,000 using higher-energy collisional dissociation.

Peptides were identified using PEAKS Studio X+ with a human UniProt database containing common contaminants. The parent and fragment mass error tolerances were 10 ppm and 0.02 Da, respectively. The enzyme was set to ‘None’ with an ‘unspecific’ digestion mode. Variable post-translational modifications (PTMs) included oxidation (+15.9949) at methionine, deamidation (+0.984) at asparagine and glutamine, acetylation (+42.0106) at the N-terminus, and cysteinylation (+119.0041) at cysteine, with a false discovery rate (FDR) of 5%. Peptide-binding motif analysis was performed using 9-mers from each sample.

The mass spectrometry proteomics data have been deposited to the ProteomeXchange Consortium via the PRIDE partner repository with the dataset identifier PXD082505 and 10.6019/PXD082505.

**HLA-B Modeling**

Structural comparisons of the HLA‑B B‑pocket region were performed in PyMOL (Schrödinger, LLC). Coordinates for HLA‑B*57:01 (PDB 5VUD) and HLA‑B*58:01 (PDB 5VWH) were downloaded from the Protein Data Bank and loaded into PyMOL. For each structure, the region surrounding residue 45 and residue 67 within the peptide‑binding groove was displayed as a close‑up view, with the HLA α1/α2 domain shown as cartoon and residues of interest rendered as sticks and color‑coded (Met45/Thr45 and Met67). Putative hydrogen‑bond/polar contacts between residue 45 and the residue‑67 region were identified using Action Find Polar contacts after selecting the relevant residues (or their local neighborhood). Distances between candidate donor–acceptor atom pairs were displayed using PyMOL distance measurements. Consistent with standard visualization criteria, strong hydrogen bonds were interpreted as < ~2.5 Å, and moderate/weaker hydrogen bonds as ~2.5–4.0 Å. Rendered images were exported directly from PyMOL for figure preparation.

**Molecular docking and structural modeling of CBZ binding**

To identify plausible CBZ binding sites within the TCR-peptide-HLA complex, we used a two‑stage docking workflow combining global site discovery with local refinement. First, DiffDock v1.1 a generative diffusion model for blind molecular docking (5,6) was used to perform a truncated version of PDB (2YPL) encompassing the TCR/HLA interface (Residues in Chains A (HLA Alpha Chain):1-181, D (TCR Alpha Chain): 2-110, E (TCR Beta Chain): 3-108) and the peptide without reliance on force‑field–based energy minimization. DiffDock was used to generate CBZ poses, using the RCSB PDB (N6W) ideal 3D conformer of CBZ and all 10 predicted poses localized to the same general region, supporting a consistent candidate binding pocket under the peptide. Because DiffDock does not output a fully minimized protein-ligand complex, direct overlays can contain residual steric clashes; accordingly, Diffdock poses were interpreted only as evidence for the binding region, not for optimized atom‑level contacts. Next, DiffDock’s consensus pocket was used to define a local docking region for Rosetta-based docking, which performs conformational search and energy optimization to refine ligand placement. Ligand docking was performed using the ROSIE RosettaLigand server (7-11) using the same protein and drug structures as for Diffdock, with the following input settings: {"angle_step": 10.1, "app": "ligand_docking", "chain": "X", "gen_conformers": true, "grid_width": 30.0, "highres_cycles": 10, "highres_repack_cycles": 3, "initial_perturb": 3.0, "move_step": 0.2, "n_ligand_conformers": 200, "nstruct": 404, "pocket_width": 7.0, "transform_cycles": 500, "use_input_position": false, "x_start": -8.219, "y_start": -31.285, "z_start": 34.944}. Rosetta docking generated multiple refined CBZ poses within the DiffDock-defined pocket; and the structure with the lowest interface delta (-14.182) and one of the lowest total scores (-620.808) was selected for visualization and contact interpretation. Overall, DiffDock was nominated as the most likely CBZ-binding region at the TCR-pMHC interface, and Rosetta-refined structures were used for final structural interpretation.

Molecular graphics for Figure 6 were generated in PyMOL (Schrödinger, LLC). Structural models and predicted carbamazepine (CBZ) poses generated as described in the docking and modeling methods were imported into PyMOL in PDB or SDF format, as appropriate. For Figure 6A, the ensemble of CBZ poses generated by DiffDock was overlaid on the same 2YPL-derived TCR–peptide–HLA input structure used for DiffDock docking. The HLA class I α1/α2 peptide-binding platform was displayed as a green cartoon, the peptide was displayed in magenta using cartoon representation, and the CBZ poses were displayed as sticks to illustrate their clustering beneath the peptide. For Figure 6B, the selected RosettaLigand output complex was visualized. This structure contained the refined CBZ pose together with the Rosetta-refined protein side-chain coordinates. The ternary complex was displayed with HLA in green, the TCR in yellow, and the peptide in magenta. CBZ was shown as pale-blue spheres to emphasize its predicted position beneath the peptide. HLA residues Tyr9, Met45, and Met67 were highlighted using sphere representations and colored pink, sky blue, and yellow-orange, respectively. TCR residue Tyr95 was highlighted in red.

For Figure 6C, TCR Vα domains were extracted from the indicated structures (PDB: 2YPL (KF11 TCR), 3VXR (3VXR (matching Va 21.01 and partial CDR3 matching MDSSYKLIF to Private TCR_1) and structurally superposed in PyMOL using standard alignment functions on Cα atoms; aligned Vα domains were shown as cartoons (KF Vα red, Private_1 Vα blue) and the conserved CDR tyrosine position was highlighted for comparison. For all panels, non-essential atoms (e.g., crystallographic waters/ions) were hidden for clarity, and final images were exported from PyMOL using ray tracing (PNG format) with consistent camera settings across conditions.

**Statistical Evaluation**

Data are presented as mean ± SD unless otherwise indicated. Flow-cytometry activation assays were performed in two independent experiments, and the figures show one representative experiment with similar results observed in the repeat experiment. Technical replicate wells within the representative experiment are displayed to show within-experiment variability but were not treated as independent biological replicates. Biological replicates were defined as independent experiments performed on separate days; for murine splenocyte APC assays, independent splenocyte preparations and/or mice were considered biological replicates where applicable. Because the main flow-cytometry figures present representative experiments, statistical comparisons were interpreted descriptively unless otherwise specified in the figure legend.

For panels comparing multiple CBZ concentrations within a single TCR-APC/HLA condition, each activation marker (PD-1, CD69, and 4-1BB) was analyzed separately by one-way repeated-measures ANOVA, followed by multiple-comparison test comparing each CBZ-treated condition with the matched 0 µg/mL CBZ control, unless otherwise indicated. For comparisons between two groups, unpaired Student’s t test was used as appropriate. When assumptions for parametric testing were not met, non-parametric tests were used where applicable. Statistical analyses were performed using GraphPad Prism 10. Statistical significance is indicated as follows: *P < 0.05, **P < 0.01, ***P < 0.001, ****P < 0.0001; ns, not significant.

**Venn diagram**

Peptide overlap across alleles was assessed in our laboratory and summarized using Venn diagrams (12).

**Supplementary Figure Legends**

Supplementary Figure 1. Validation of HLA expression on APCs and engineered T-cell lines used for functional assays. (A) Flow cytometric confirmation of human CD8 and murine TCR expression on parental TG40 cells and TCR-transduced TG40 cells. (B) Representative flow cytometry histograms showing surface expression of transfected HLA class I molecules on monoallelic 721.221 APCs expressing HLA-B*15:02, HLA-B*57:01, or HLA-B*58:01 compared with 721.221-Null cells. (C) Comparable HLA class I surface expression on 721.221-HLA-B*58:01 and HLA-B*58:01(TE🡪MA).(D) Surface staining of splenic B cells (CD3^-^CD19^+^B220^+^) from WT, HLA-B*15:02 Tg, HLA-B*15:02 Tg/MHC I KO, HLA-B*57:01 Tg, and HLA-B*57:01 Tg/MHC I KO mice. Representative flow-cytometry histogram overlays are shown with fluorescence intensity on a logarithmic x-axis and event counts on a linear y-axis normalized to mode using the FlowJo Modal normalization function.

Supplementary Figure 2. Representative flow-cytometry histograms of activation marker upregulation in public and private TCR lines cultured with monoallelic 721.221 APCs expressing HLA-null, HLA-B*15:02, HLA-B*57:01, or HLA-B*58:01 and treated with CBZ (0 (no-drug/no-vehicle), 25, or 50 µg/mL). Representative flow-cytometry histogram overlays are shown with fluorescence intensity on a logarithmic x-axis and event counts on a linear y-axis normalized to mode using the FlowJo Modal normalization function. N = 3 (technical replicates), Bars show mean ± SD of technical replicate wells from one representative experiment; experiments were performed twice independently with similar results (biological replicates).

Supplementary Figure 3. Representative flow-cytometry histograms of CBZ responses using HLA-transgenic murine splenocyte APCs. Public and private TCR lines were co-cultured with splenic APCs from WT mice or HLA-B*15:02 Tg/MHC I KO or HLA-B*57:01 Tg/MHC I KO mice and treated with CBZ (0 (no-drug/no-vehicle), 25, or 50 µg/mL). Representative flow-cytometry histogram overlays are shown with fluorescence intensity on a logarithmic x-axis and event counts on a linear y-axis normalized to mode using the FlowJo Modal normalization function. N = 3 (technical replicates), Bars show mean ± SD of technical replicate wells from one representative experiment; experiments were performed twice independently with similar results (biological replicates).

Supplementary Figure 4. Sequence alignment of HLA-B*15:02, HLA-B*57:01, and HLA-B*58:01 heavy chains. Amino-acid alignment of HLA-B*15:02:01:01, HLA-B*57:01:01:01, and HLA-B*58:01:01:01 highlights residues 45/46 in the B pocket.

Supplementary Figure 5. Peptide identification yield and length-stratified self-peptide overlap among HLA-B immunopeptidomes. Total unique peptides and shared peptides are shown for HLA-B*15:02, HLA-B*57:01, HLA-B*58:01, and HLA-B*58:01(TE🡪MA).

Supplement Table 1. Shared self‑peptide sequences across HLA‑B immunopeptidomes.
Peptide sequences (single‑letter code) that overlap between the indicated HLA‑B allotypes are listed for pairwise comparisons (HLA‑B*57:01 [5701] vs HLA‑B*15:02 [1502]; HLA‑B*58:01 [5801] vs HLA‑B*15:02; HLA‑B*58:01(TE🡪MA) [58:01_MA] vs HLA‑B*15:02) and for three‑way intersections (HLA‑B*57:01/HLA‑B*15:02/HLA‑B*58:01 and HLA‑B*57:01/HLA‑B*15:02/HLA‑B*58:01(MA)). Numbers shown under each header indicate the total number of shared peptides identified in each overlap set.

1.	Zumla A, Marguerie C, So A, Yokoyama WM, Saito T, Batchelor JR, et al. Co-expression of human T cell receptor chains with mouse CD3 on the cell surface of a mouse T cell hybridoma. J Immunol Methods. 1992;149(1):69-76.

2.	Ko TM, Chung WH, Wei CY, Shih HY, Chen JK, Lin CH, et al. Shared and restricted T-cell receptor use is crucial for carbamazepine-induced Stevens-Johnson syndrome. J Allergy Clin Immunol. 2011;128(6):1266-76 e11.

3.	Pan RY, Chu MT, Wang CW, Lee YS, Lemonnier F, Michels AW, et al. Identification of drug-specific public TCR driving severe cutaneous adverse reactions. Nat Commun. 2019;10(1):3569.

4.	Mifsud NA, Illing PT, Lai JW, Fettke H, Hensen L, Huang Z, et al. Carbamazepine Induces Focused T Cell Responses in Resolved Stevens-Johnson Syndrome and Toxic Epidermal Necrolysis Cases But Does Not Perturb the Immunopeptidome for T Cell Recognition. Front Immunol. 2021;12:653710.

5.	Corso G, Stärk H, Jing B, Barzilay R, Jaakkola T. Diffdock: diffusion steps, twists, and turns for molecular docking. International Conference on Learning Representations (ICLR). 2023.

6.	Corso G, Deng A, Polizzi N, Barzilay R, Jaakkola T. Deep Confident Steps to New Pockets: Strategies for Docking Generalization. International Conference on Learning Representations (ICLR). 2024.

7.	Davis IW, Baker D. RosettaLigand docking with full ligand and receptor flexibility. J Mol Biol. 2009;385(2):381-92.

8. Deluca, S., Khar, K., Meiler, J. Fully Flexible Docking of Medium Sized Ligand Libraries with RosettaLigand. PLoS ONE. 2015; 10(7): e0132508. doi:10.1371/journal.pone.0132508

9. Combs, S. A., Deluca, S. L., DeLuca, S. H., Lemmon, G. H., Nannemann, D. P., Nguyen, E. D., et al. Small-molecule ligand docking into comparative models with Rosetta. Nature Protocols. 2013; 8(7), 1277–1298. doi:10.1038/nprot.2013.074

10. Kothiwale, S., Mendenhall, J.L., Meiler, J. BCL::Conf: small molecule conformational sampling using a knowledge based rotamer library. J. Cheminform. 2015; 7, 47. doi:10.1186/s13321-015-0095-1

11. Lyskov S, Chou FC, Conchúir SÓ, Der BS, Drew K, Kuroda D, Xu J, Weitzner BD, Renfrew PD, Sripakdeevong P, Borgo B, Havranek JJ, Kuhlman B, Kortemme T, Bonneau R, Gray JJ, Das R., "Serverification of Molecular Modeling Applications: The Rosetta Online Server That Includes Everyone (ROSIE)". PLoS One. 2013 May 22;8(5):e63906. doi: 10.1371/journal.pone.0063906. Print 2013.

12.	Oliveros JC. Venny. An interactive tool for comparing lists with Venn’s diagrams 2007 [Available from: <https://bioinfogp.cnb.csic.es/tools/venny/index.html>.
